# A Review of Imaging Methods to Assess Ultrasound-Mediated Ablation

**DOI:** 10.34133/2022/9758652

**Published:** 2022-05-02

**Authors:** Brett Z. Fite, James Wang, Pejman Ghanouni, Katherine W. Ferrara

**Affiliations:** Department of Radiology, Stanford University, Palo Alto, CA 94305, USA

## Abstract

Ultrasound ablation techniques are minimally invasive alternatives to surgical resection and have rapidly increased in use. The response of tissue to HIFU ablation differs based on the relative contributions of thermal and mechanical effects, which can be varied to achieve optimal ablation parameters for a given tissue type and location. In tumor ablation, similar to surgical resection, it is desirable to include a safety margin of ablated tissue around the entirety of the tumor. A factor in optimizing ablative techniques is minimizing the recurrence rate, which can be due to incomplete ablation of the target tissue. Further, combining focal ablation with immunotherapy is likely to be key for effective treatment of metastatic cancer, and therefore characterizing the impact of ablation on the tumor microenvironment will be important. Thus, visualization and quantification of the extent of ablation is an integral component of ablative procedures. The aim of this review article is to describe the radiological findings after ultrasound ablation across multiple imaging modalities. This review presents readers with a general overview of the current and emerging imaging methods to assess the efficacy of ultrasound ablative treatments.

## 1. Introduction

The goal of this review is to characterize imaging methods for monitoring and assessing ultrasound-mediated ablation. The imaging methods are organized in descending order of current clinical application, and we include some promising emerging technologies. Before discussing imaging modalities, we will provide a brief overview of the types and mechanisms of ultrasound ablation.

### 1.1. Thermal Ablation.

High intensity focused ultrasound (HIFU) is a versatile treatment modality. HIFU is noninvasive and can be steered in real time. The ultrasound parameters employed dictate how the ultrasound wave interacts with tissue (e.g., absorption or generation of cavitation bubbles), which in turn cause bioeffects such as heating or mechanical destruction, generating varied types of transition zones and mechanisms of tissue death. With a low mechanical index, mechanical effects are minimized, and the targeted tissue absorbs acoustic energy and primarily thermal effects are generated. Thermal ablation is usually induced with a low mechanical index and high duty cycle, resulting in high time-averaged power. Under conditions for thermal ablation, HIFU produces a region of coagulative necrosis approximating the focal spot volume, which is dependent on transducer geometry and central frequency. With a clinical system operating in the low megahertz or hundreds of kilohertz range, this often exceeds 1 × 1 × 7 mm^3^, but can be significantly smaller with an optimized small animal HIFU system. The ablated region can be further increased by scanning the beam (e.g., in a circular pattern [1]).

Thermal ablation occurs when sufficient thermal dose is delivered to the target tissue to rapidly induce coagulation and necrosis (50–100°C). The extent and rate of tissue damage varies due to such factors as the amount of energy and rate of energy application, the energy absorption and vascularity of the tissue, and the duration of application. Thus, thermal ablation benefits from both real-time monitoring and posttreatment quantification of the extent of tissue death.

### 1.2. Mechanical Destruction.

At higher pressures, HIFU exerts mechanical effects through the production of cavitation bubbles (histotripsy) or microscopic boiling bubbles (boiling histotripsy) [2, 3]. In histotripsy, lower frequencies and high pressures are used for very short durations, often only a single cycle (on the order of microseconds), which causes acoustic cavitation from gas dissolved in tissue. Boiling histotripsy employs several cycles (on the order of milliseconds) that initially cause a microscopic region to boil, and the subsequent cycles will cause the boiling bubble to oscillate and finally collapse. Although thermal HIFU generates a narrow transition zone, mechanical disruption from either boiling histotripsy or histotripsy typically results in homogenization and eventual resorption of the liquefied tissue with an even more narrow transition region than thermal HIFU [4, 5]. A narrower transition zone is especially desirable in cases where a tumor may be close to critical tissue structures. An additional advantage of mechanical disruption is its ability to be applied in highly vascularized tissue where thermal ablation may be limited due to the heat sink effect.

In addition to generating cavitation and boiling bubbles to induce tissue destruction, exogenous microbubbles or liquid perfluorocarbon droplets can be used with lower pressures to mechanically destroy tissue [6]. Microbubbles are gas bubbles (typically perfluorocarbons or sulfur hexafluoride) enclosed within a stabilizing shell, which is often lipid or protein but can be composed of polysaccharide or other polymers. Although this method is not employed clinically, it has been investigated to enhance the ablative effect of ultrasound in preclinical models.

## 2. Imaging Techniques

### 2.1. Overview of Imaging Modalities.

The physiologic response to HIFU depends on the specific parameter set used (e.g., central frequency, pressure, and duty cycle) but can be divided roughly into two regimes: (1) thermal and (2) mechanical. For thermal ablation, coagulative necrosis primarily dictates the imaging features. Immediately following thermal ablation, reactive hyperemia is present in the tissue adjacent to the ablated region due to obstruction of blood flow in the ablated tissue. Mechanically ablative regimes rapidly destroy vasculature, leading to significantly decreased perfusion within the ablated region. Reactive hyperemia and inflammation are less common with mechanical ablation due to the very narrow transition zone between ablated and viable tissue. Here, we will examine imaging modalities from the most frequently clinically utilized to emerging techniques. Magnetic resonance imaging (MRI) is the most widely employed imaging modality clinically for the guidance and assessment of HIFU ablation due to its excellent soft tissue contrast and ability to map temperature in real time. X-ray computed tomography (CT) is the second most widely used modality for ablation assessment due to its ability to rapidly acquire volumetric datasets. A summary of the imaging modalities used for assessment of thermal and mechanical HIFU ablation and the radiological findings is provided in Table 1. This summary table of ablation and imaging is structured according to MRI, CT, US, and PET imaging.

### 2.2. Cross Modality Measures

#### 2.2.1. RECIST.

Morphological measurements have been used to assess ablation via registration of pre and postablation images. However, dimensional criteria, such as the Response Evaluation Criteria in Solid Tumors (RECIST) [23, 24], while considered the gold standard of response assessment in solid tumors, cannot be accurately applied shortly after thermal ablation to quantify viable tumor. Due to inflammation and hemorrhage, lesion size may initially appear to increase, and prognostic changes in morphology may not be apparent for up to 6–12 months. For mechanical HIFU, there is markedly less inflammation as most of the tissue is fragmented and liquefied with only a very thin border between treated and healthy tissue. However, hemorrhage may occur in highly vascularized tissues (e.g., liver) which may confound morphological evaluation of treatment efficacy. The shortcomings of morphological evaluation of response are well-documented for novel therapeutics, especially immunotherapies, and more emphasis is being placed on functional and metabolic biomarkers of response [25–27] such as alterations in perfusion, spectroscopic and other methods to examine metabolic activity, and radiomic techniques that include multiple quantitative imaging features.

#### 2.2.2. mRECIST.

RECIST was initially adopted to evaluate the response of solid tumors to cytotoxic agents. However, subsequent advances in therapies including focal ablation, targeted therapies, immunotherapies, and locoregional therapies have led to the development of a modified RECIST (mRECIST) assessment of response. RECIST criteria measure dimensions, mRECIST criteria measure only the enhanced (viable) portion of lesions. Compared to RECIST, mRECIST is a more accurate predictor of response following thermal ablation [28]. Indeed, for HIFU ablation in the liver for primary and metastatic disease in 275 patients, the response as evaluated by mRECIST was found to be an independent risk factor for overall survival while response evaluated with RECIST criteria was not statistically correlated with overall survival [29] suggesting that modified morphological criteria to assess disease progression are needed to properly evaluate the effects of ablative therapies.

#### 2.2.3. Nonperfused Volume.

Contrast-enhanced scans, used to visualize perfused tissue, can detect coagulative necrosis which is often observed as a rim of greater enhancement around the nonenhancing (due to destruction of vasculature) ablated zone regardless of the modality used. Perfusion can be assessed with all commonly used imaging modalities when a contrast agent is used, but the sensitivity varies by modality. The nonperfused volume is often assessed immediately after HIFU to gauge ablation margins; however, the immediate inflammatory reaction, especially when accompanied by reactive hyperemia, may obscure characteristics of viable tumor.

## 3. Magnetic Resonance Imaging

The ability to combine assessments of temperature, perfusion, tissue elasticity, and diffusion within an imaging and treatment session leads to the preference of many clinicians to guide therapy with MRI. MRI is the most commonly used modality for monitoring thermal ablation because of its sensitivity to temperature changes even beyond the temperature at which coagulation occurs (unlike ultrasound) and, while CT has similar to superior temporal resolution, MRI does not use ionizing radiation, and thus, real-time scans can be performed continuously without regard to radiation dose limits. The excellent soft tissue contrast of MRI combined with its prevalence of use for procedure monitoring makes it the most commonly used modality for postprocedure assessment.

### 3.1. MRI Thermometry and Real-Time MRI.

Magnetic resonance thermometry (MRT) based on the proton resonance frequency shift (PRFS) method [30, 31] is the most widely used real-time guidance method for thermally ablative HIFU. PRFS-based MRT can be employed in 2D with a single slice covering the HIFU focal region, in multiple slices, either parallel or orthogonal, surrounding the focal plane, or volumetrically. However, volumetric imaging has not as yet been employed clinically. The ability to map temperature volumetrically in real time is an advantage of MR guidance of HIFU. In addition, PRFS-based MRT exhibits a linear dependence on temperature, is consistent across aqueous tissue types, and provides accurate relative temperature measurements even in coagulated tissue. However, because the PRFS method is based on the resonance frequency shift of water protons, it can be challenging to apply in tissues with high-fat content. This can be overcome with other MRT techniques as nearly all tissue parameters measurable with MR exhibit a dependence on temperature. Proton density, T_1_, T_2_, diffusion, and susceptibility have all demonstrated the ability to quantify temperature without exogenous contrast [31]; however, these techniques often require longer scan times and/or a higher signal-to-noise ratio (SNR) than PRFS-based thermometry. MRT techniques other than PRFS have not been utilized clinically.

Real-time MRI has also been explored for the near real-time monitoring of histotripsy by sensitizing bipolar gradients to the lifespan of the induced bubble cloud [32], which permits detection of the bubble cloud at the onset of histotripsy. Previous methods have focused on incoherent water motion induced by the bubble cloud. The primary drawback of the latter method is that it works best in previously homogenized tissue versus during the expansion stage of the bubble cloud.

### 3.2. MR Acoustic Radiation Force Imaging (MR-ARFI).

MR-ARFI uses micron-scale ultrasonic displacement of a small focal region within the MR image to assess the tissue displacement [33–35]. Motion encoding gradients that are synced with the HIFU source are incorporated in the MRI sequence to encode the motion into the phase data. This technique has the advantages of localizing the beam and providing an estimate of the ultrasound pressure deep within the body without an increase in temperature [36], thus facilitating optimization of the treatment. Since thermal ablation increases the tissue stiffness, MR-ARFI can provide feedback on the ablation treatment in real time. The time required to estimate the stiffness of each region can limit the utility but faster sequences can be implemented [37]. Although not in general clinical use, transient MR elastography is another method with the ability to estimate the extent of ablation over a wider region of tissue [38].

### 3.3. MRI for Postprocedure Assessment.

MRI provides excellent soft tissue contrast through a variety of mechanisms. Endogenous contrast mechanisms available in MRI include differences in the longitudinal relaxation time (T1), transverse relaxation time (T2), proton density (PD), and local changes in the diffusion of water. Additionally, exogenous contrast agents may be used, most often gadolinium chelates or iron nanoparticles. The most common MR method to assess ablation is perfusion-based, contrast-enhanced fat-suppressed T1w imaging. MRI provides excellent monitoring of both the immediate treatment response [39–42] and local disease progression on subsequent follow-up imaging. MR-based methods are especially advantageous following HIFU since HIFU procedures are often MR guided. In this section, we will provide an overview of MR methods used to assess ablation from most commonly employed to emerging techniques.

**Figure 1:**
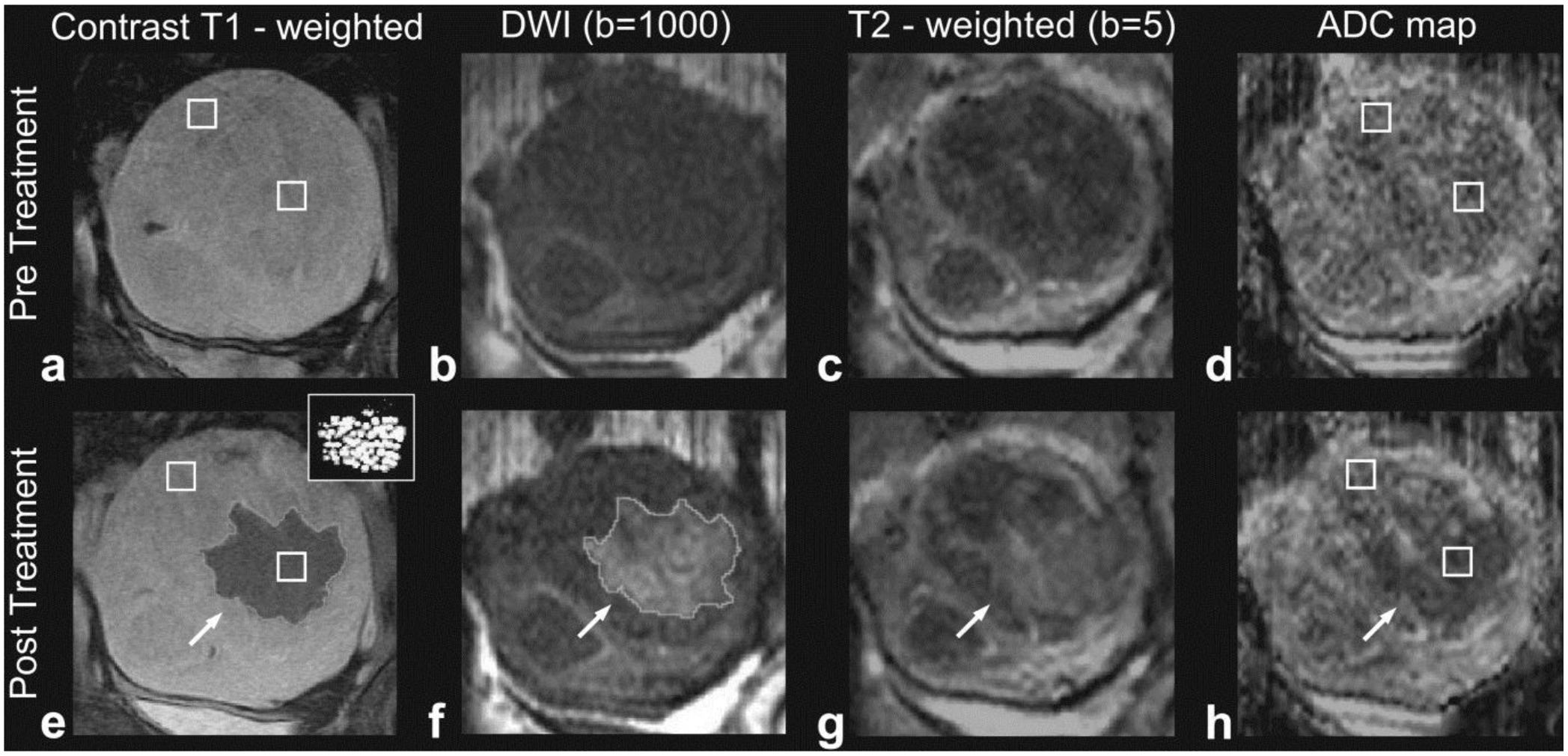
Multiple parameter MRI assessment of thermal HIFU ablation of uterine fibroids. Patient 31 (52 years old, intramural leiomyoma). Coronal images at the treatment plane pre- and post-HIFU ablation. (a) Contrast-enhanced T1-weighted image acquired pretreatment. (b) DW image acquired pretreatment. (c) T2-weighted image acquired pretreatment. (d) ADC map pretreatment (ADC value: 1287 ± 258 × 10^−6^ mm^2^/s). (e) Contrast-enhanced T1-weighted image acquired posttreatment. The area of the resulting nonperfused region (demarcated with a light grey line in pane e) was 20.8 cm^2^. The thermal dose estimate calculated from the temperature maps is shown in the inset figure, at the upper right corner. Areas that reached a thermal dose of 240 min are grey, and areas that reached a thermal dose of 18 min are white. The area of the region with a thermal dose exceeding 18 min at 43°C was 14.7 cm^2^. (f) DW image acquired posttreatment. The area of the resulting hyperintense region (outlined with a light grey line in pane f) was 22.8 cm^2^. (g) T2-weighted image acquired posttreatment, the affected area appears hyperintense (h) ADC map posttreatment, the affected area appears hypointense (ADC value: 923 ± 175 × 10^−6^ mm^2^/s). Figure adapted from [8].

By far, the most common MR method to assess ultrasound ablation is gadolinium contrast-enhanced T1-weighted (T1w) imaging. In addition to comparing to pretreatment contrast-enhanced T1w images (Figure 1(a)), postablation regions visualize the region of coagulative necrosis generated by ablation as nonenhancing. This region is sometimes surrounded by an enhancing rim [7] of reactive hyperemia that is homogenous and encompasses the circumference of the ablation zone. Residual viable tumor will typically present with nodular enhancement around the periphery of the ablated region. Contrast-enhanced MRI is about twice as sensitive to residual disease than contrast-enhanced CT [43]. The nonenhancing central ablated region in a murine breast tumor was highly correlated to lethal thermal dose (CEM 240) [44].

While contrast-enhanced T1w imaging is the preferred method for clinical assessment of HIFU ablation, it is typically performed only at the end of thermal HIFU procedures due to acceleration of gadolinium dechelation kinetics, and thus a decrease in dissociation half-life with increasing temperature [45, 46]. Therefore, MR protocols relying on endogenous contrast are advantageous as they may be repeated as needed throughout a procedure. The most commonly used endogenous contrast MR method is diffusion-weighted imaging (DWI), which generates contrast through local differences in the diffusion of water by applying a diffusion-encoding gradient during a scan. A DWI scan’s *b* value measures the degree of diffusion weighting in the scan. Higher *b* values (Figure 1(b)) have a greater degree of diffusion weighting than lower *b* values (Figure 1(c)). More quantitatively, a series of diffusion-weighted images with varying diffusion encoding gradient strength or duration can be used to estimate the apparent diffusion coefficient (ADC) for each voxel via fitting to a single exponential, generating an ADC map (Figure 1(d)). DWI and ADC maps have been explored to evaluate the effects of ablation. Ablative treatments are expected to reduce cellularity and perfusion while increasing necrosis. These competing effects can make diffusion-based contrast difficult to interpret [47]. However, several studies have reported a decrease in tissue ADC following ablation in the prostate [48–50], uterine fibroids [8, 9, 47, 51], and brain [52–54]. The treated lesion size, as measured on diffusion MRI, has been found to correlate well with contrast-enhanced T1w images (Figure 1(e)) and histopathology. Higher *b* value diffusion scans delineate HIFU ablation margins (Figure 1(f)) more clearly than lower *b* value scans (Figure 1(g)) and may sometimes be easier to interpret than an ADC map (Figure 1(h)).

**Figure 2:**
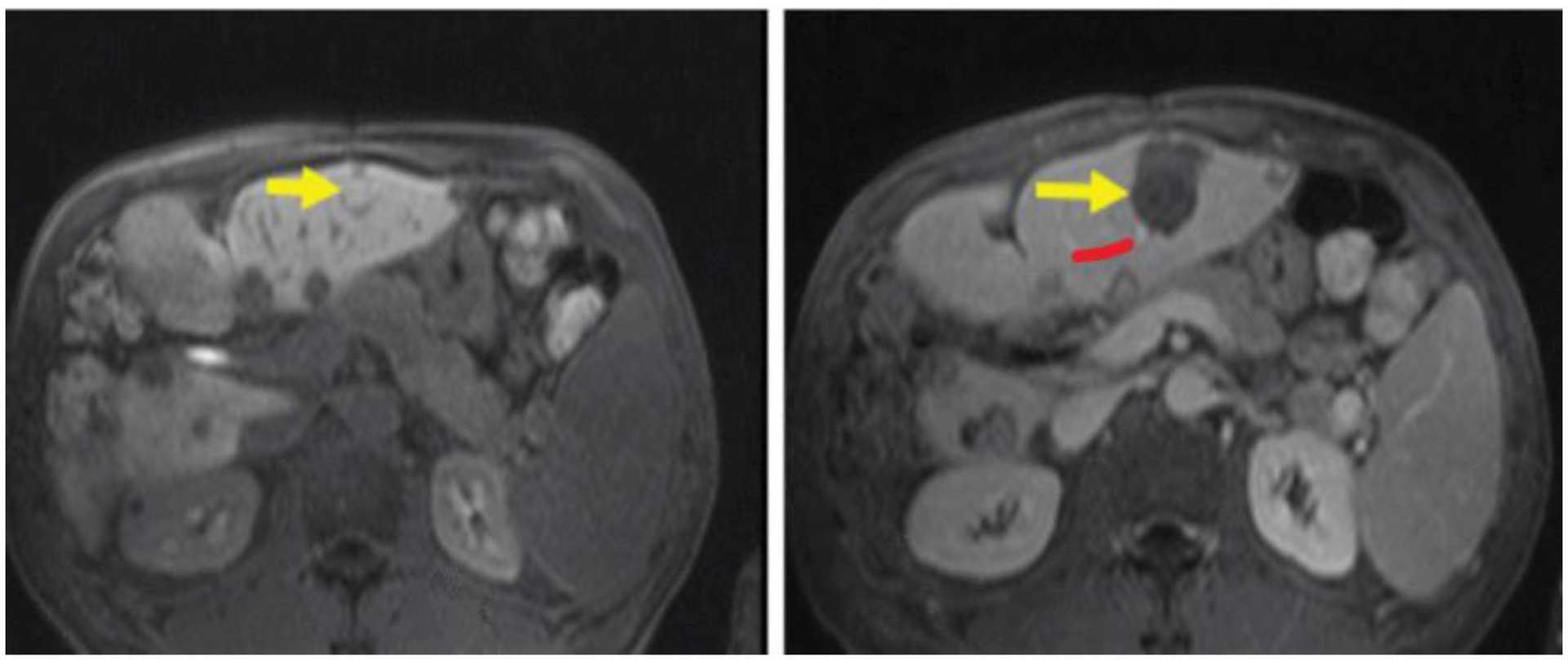
Posthistotripsy contrast-enhanced T1w MRI. MRI of the targeted tumor 5 mm adjacent to hepatic vessel branch (red arrow) before (a) and after (b) histotripsy procedure (yellow arrow). Figure adapted from [10].

Contrast-enhanced T1w imaging has also been used to evaluate the effects of histotripsy. While histotripsy does not produce the coagulative necrosis associated with thermal HIFU, the process of homogenization is anticipated to disrupt vascular supply to the treatment zone. Similar to thermal HIFU, compared to treatment planning images (Figure 2(a)), posthistotripsy contrast-enhanced T1w MRI reveals a nonperfused volume corresponding to the mechanically disrupted region of the liver.

**Figure 3:**
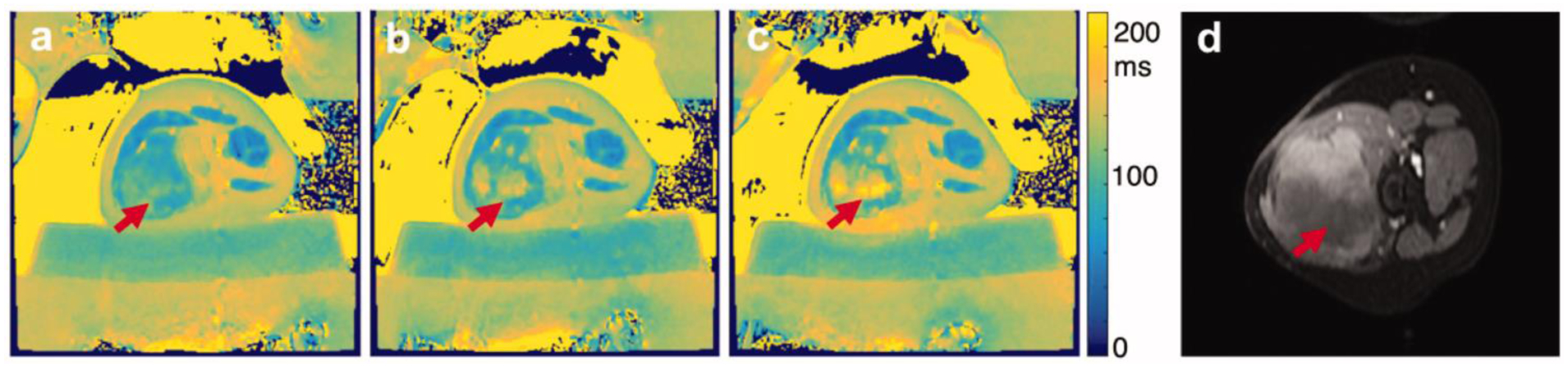
MRI T2 mapping pre and postthermal HIFU ablation desmoid tumors. Pre and postaxial T2 maps (a–c) of one slice for a thermal HIFU treatment. (a) demonstrates the tissue before sonication treatment. (b and c) demonstrate increased T2 value over the course of the treatment duration. (d) postcontrast image of same location. Patient was repositioned, but the arrows indicate the ROI. T2 maps were generated in MATLAB. Figure adapted from [11].

Non-contrast-enhanced T1w images have been used to identify ablated regions within minutes with the hyperintense regions correlating with histopathology [55–57]. On T2w images, the ablated region may sometimes appear hypointense [39, 58] or present with a hyperintense [59] rim compared to surrounding tissue. Hyperintensity on unenhanced T1w imaging and hypointensity on T2w images compared to surrounding parenchyma is observed for thermally ablated regions in the liver [60] and kidney [61]. Discrete regions of T2 hyperintensity may indicate the presence of viable tumor but may also be caused by the initial inflammatory response to thermal injury. However, in desmoid tumors, employing a T2 mapping protocol has shown a progressive increase in T2 from pretreatment (Figure 3(a)) to the early stages of HIFU ablation (Figure 3(b)) through the late stages of the HIFU treatment (Figure 3(c)), and the regions of increased T2 correspond to areas within the nonperfused volume as assessed on contrast-enhanced T1w imaging (Figure 3(d)).

**Figure 4:**
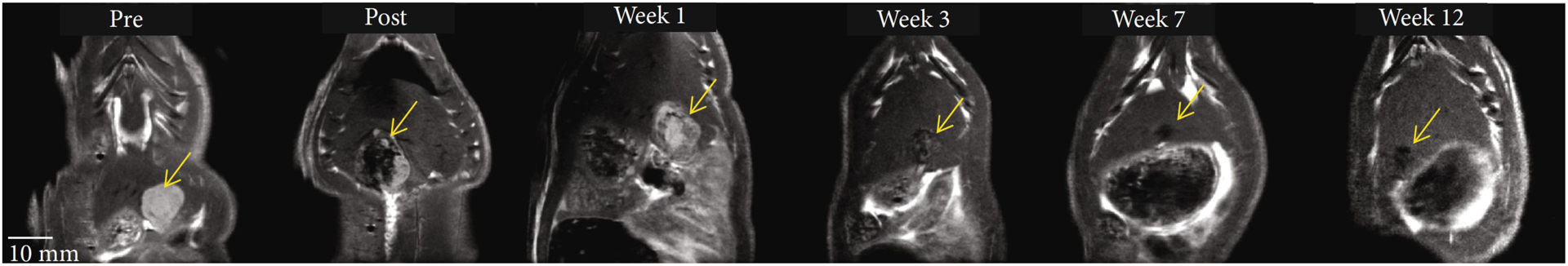
MRI T2-weighted imaging pre and posthistotripsy in a preclinical model of liver cancer. Tumor response to histotripsy on T2-weighted MRI. The size and appearance of tumor (yellow arrow) in response to complete and partial histotripsy ablation are observed at different timepoints. Figure adapted from [12].

After boiling histotripsy, a successfully mechanically disrupted region may appear hyperintense on T2w images, with the possible presence of a hypointense rim surrounding it [5], but the appearance of the mechanically disrupted lesion will vary depending on how long after HIFU the tissue is imaged. Conversely, in preclinical tumor models, the region treated with histotripsy may appear hypointense shortly after the procedure (Figure 4). The T2w hypointensity seen shortly after histotripsy evolves to become hyperintense after which the intensity gradually decreases over time. T2* non-contrast imaging has also been explored to visualize thermally ablated regions. Coagulative necrosis and other changes in tissue properties from thermal ablation can create susceptibility differences [62, 63], which appear as hypointense regions on a T2* weighted image.

A promising diffusion-based imaging technique, which is not as yet widely utilized clinically is intravoxel incoherent motion. Currently, this technique has only been applied after radiofrequency ablation; however, given the relatively wide use of DWI in the clinical setting to assess HIFU, this technique has the promise of rapid translation for HIFU peri and postprocedure assessment. In contrast to the generation of a traditional ADC map, intravoxel incoherent motion [64] attempts to separate the contributions of water diffusivity and tissue perfusion via fitting a series of images acquired at different *b* values to a biexponential instead of a single exponential. Intravoxel incoherent motion on DWI sequences also correlates with therapeutic response immediately following thermal ablation despite a lack of correlation with perfusion-based metrics [65]. Of all intravoxel incoherent motion parameters, ADC_total_ was the best discriminator between necrotic and viable tissue postthermal ablation but could not reliably discriminate between necrotic and inflammatory tissue [66]. During the early postablation stage, there is significant inflammation which may be confused with necrosis at the rim of the ablated zone where ADC_total_ to be used as the sole discriminator. However, a second parameter, *f* , representing the relative weighting of the microperfusion and water diffusion components within each voxel, showed high discriminatory power to differentiate between necrotic and inflammatory regions postablation.

**Figure 5:**
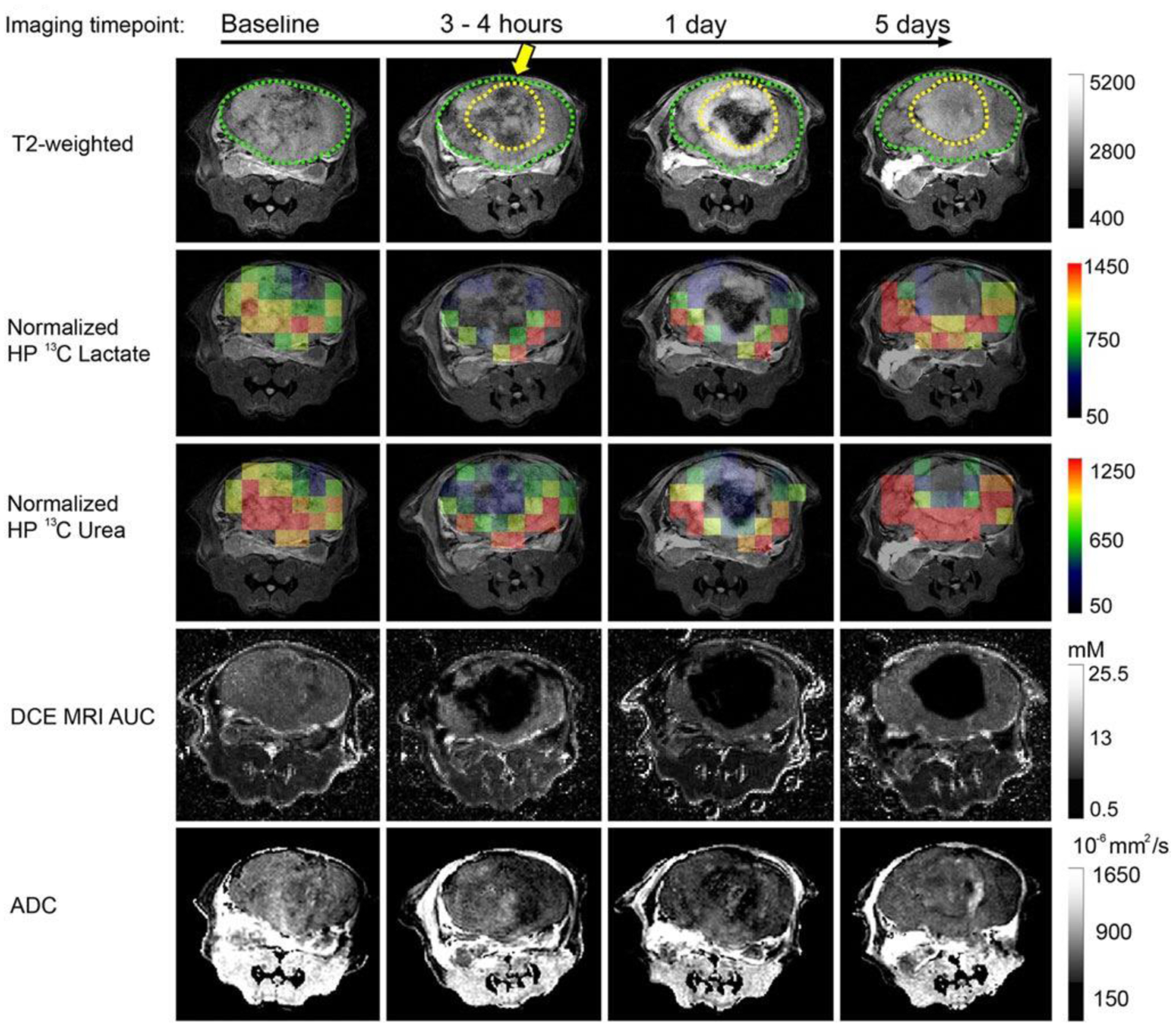
Multiparametric and heteronuclear MRI imaging pre and postthermal HIFU in a preclinical mouse model. T2-weighted images pre- and post-HIFU thermal ablation treatment, overlaid HP ^13^C lactate and ^13^C urea images on the T2-weighted images, DCE MRI AUC, and ADC. The position of the HIFU applicator is indicated by the yellow arrow. The region of ablation is demarcated by a dashed yellow line. Figure adapted from [13].

An emerging MR technique for ablation assessment is heteronuclear MRI, which, in addition to providing morphological information, can be utilized to provide metabolic information, which can probe the functional state of tissue. The relatively low sensitivity of MRI can be overcome through hyperpolarization, which increases the signal to noise by ~10^4^ – 10 [5]. Hyperpolarized MRI, especially ^13^C and ^15^N, have been employed to probe multiple functional pathways simultaneously. Recently, a combination of hyperpolarized ^13^C pyruvate and ^13^C urea has been applied to assess HIFU ablation in a mouse model of prostate cancer [13]. ^13^C urea, which is metabolically inactive, serves as perfusion contrast, while ^13^C pyruvate is utilized to measure metabolic activity [67]. This method demonstrated good discrimination between sublethal and lethal thermal doses within several hours of treatment without the need for gadolinium contrast. However, the spatial resolution of MR spectroscopic imaging protocols is still limited. Furthermore, imaging must be carried out rapidly because the increased signal derived from hyperpolarization decays at the spin-lattice relaxation rate, R_1_. Nevertheless, this is sufficient for most imaging studies tracking the initial probe and any of its metabolites. Currently ^13^C MRI has been used to assess HIFU ablation only in preclinical models (Figure 5), but its ability to combine functional and metabolic information with perfusion-based metrics is promising.

### 3.4. Summary of MRI.

MRI offers a wide variety of techniques for temperature measurement and has numerous contrast mechanisms for postprocedure assessment. In current clinical practice, thermal ultrasound ablation is monitored almost exclusively with MR thermometry based on the PRFS method. Contrast-enhanced T1w MRI is the most common protocol for postprocedure assessment of nonperfused, ablated regions. However, techniques such as diffusion-weighted imaging are increasingly being used clinically for intraprocedural assessment prior to a final contrast-enhanced T1w scan. Contrast-enhanced T1w MRI is also used for postprocedure assessment of histotripsy, with mechanically disrupted lesions appearing as nonperfused regions.

## 4. Computed Tomography

X-ray CT has the potential to guide thermal HIFU therapies, and CT-based thermometry has been explored. With increasing temperature, tissue density will correspondently decrease due to thermal expansion. This translates into a reduction of the attenuation coefficient and thus a decrease in measured Hounsfield units (HU) of ~0.5 HU per °C [68]. Given that typical noise levels on clinical scanners range from 3 to 6 HU [69], this is sufficient temperature resolution for thermal HIFU but lacks the temperature SNR achieved with MR thermometry. Additionally, CT has ample temporal resolution to guide ablative therapies in real time. To our knowledge, real-time CT has not been employed for guidance of nonthermal HIFU therapies. Moreover, due to the increased radiation dose required for real-time monitoring, CT guidance of HIFU, either thermal or nonthermal/mechanical, is not employed clinically.

**Figure 6:**
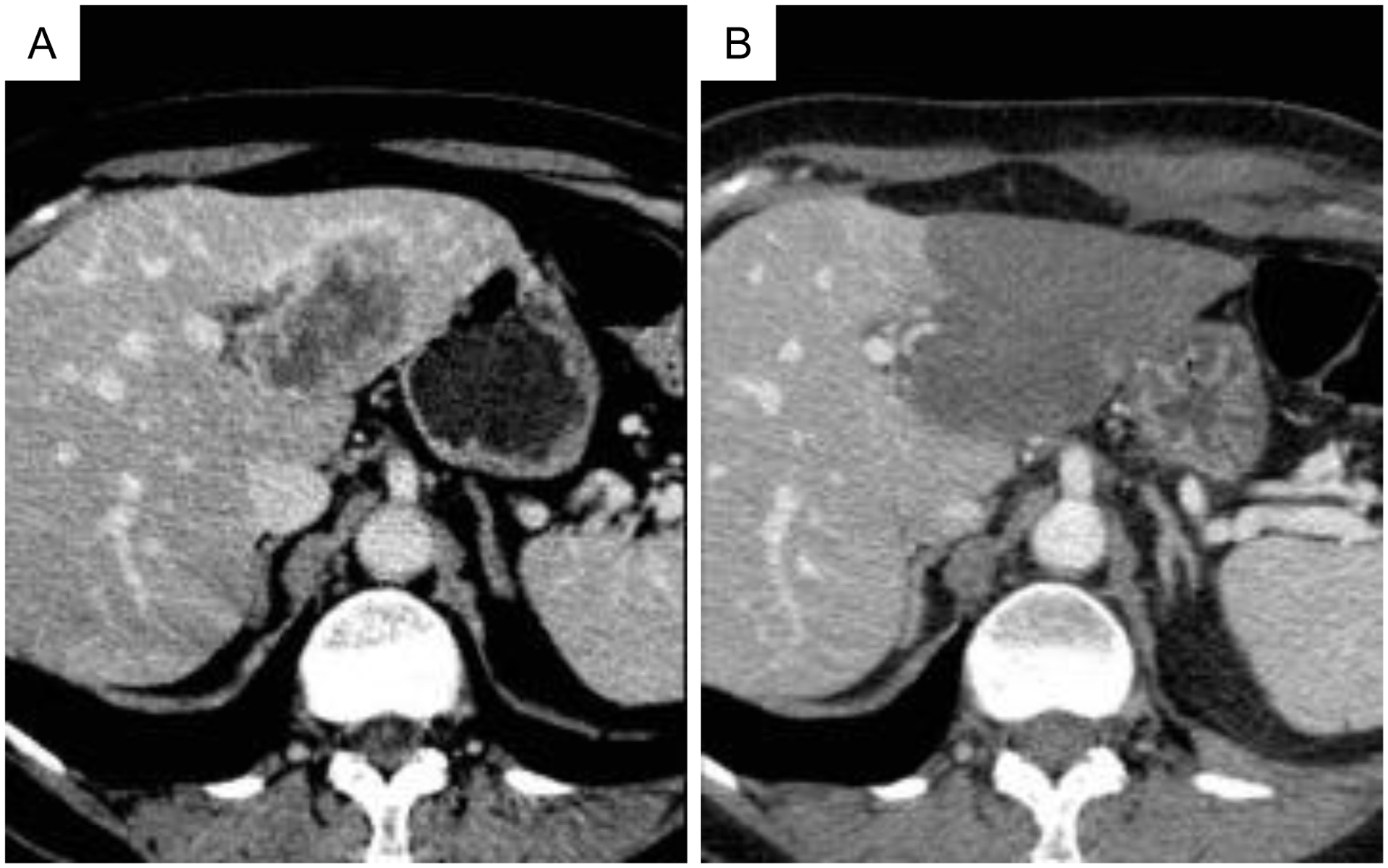
CT imaging pre and postthermal HIFU treatment of the liver. (a) Computed tomography (CT) scan of segment II–III liver metastasis from breast cancer. (b) Contrast-enhanced CT image obtained 24 h following high-intensity focused ultrasound (HIFU) ablation demonstrates a low-attenuating nonenhancing area that has replaced the tumor. No residual tumor enhancement is identified. Figure from [15].

However, CT is relatively widely employed for postprocedure assessment of thermal HIFU ablation due to its speed and availability. Similar to MRI, a contrast-enhanced scan that assesses perfusion is the most common CT technique employed to assess both thermal and mechanical HIFU ablation since both ablative techniques rapidly disrupt vasculature. Preprocedure CT images visualizing the tumor to be ablated (Figure 6(a)) can be compared to postprocedure contrast-enhanced scans (Figure 6(b)) to quantify the nonperfused volume and assess ablation margins. On immediate post-HIFU (both mechanical and thermal) ablation contrast-enhanced CT, the ablated zone appears as a nonenhancing region. Post-thermal HIFU, the ablated lesion also presents as low attenuation [14, 70, 71]. The ablated lesion is nonenhancing due to the destruction of vasculature due to thermal damage. The nonperfused region is sometimes surrounded by an enhancing rim from reactive hyperemia after thermal ablation. If present, the enhancing rim appears shortly after ablation and resolves within several days to, sometimes weeks. The rim surrounding the ablated, nonperfused volume may vary in thickness. The enhancing rim is most commonly observed on arterial phase images but not exclusively so. However, an enhancing rim that is irregular in contour or has discrete nodules may be indicative of residual tumor [72] and differentiating residual tumor from hyperemia may be challenging at these early stages. On longer term follow-up (>3 months), thermally ablated regions remain nonenhancing. Presence of a nodular enhancing rim at this stage is suggestive of recurrence, as is an increase in lesion size. Subtraction images made from multiphase or pre and postthermal ablation contrast-enhanced CT images improved correlation of lesion size with histological analysis [73].

In the absence of contrast, thermally ablated regions appear as low density regions with the possibility of a core of high attenuation [72]. In the kidney, thermally ablated lesions have higher attenuation (~40 HU) than the surrounding parenchyma [61]. The increased attenuation of the thermally ablated region remains consistent for up to 24 months. However, in the liver, thermal ablation has been reported to decrease attenuation compared to surrounding parenchyma with the maximum difference between the two occurring at 8 min postablation with a difference of ~22 HU. The presence or absence of bubbles has no observable difference on the decreased attenuation [60]. There is variability in postthermal ablation presentation between tissue types on unenhanced CT.

The appearance of the ablated region varies by size as well. Thermally ablated renal masses ≤ 3 cm^3^ increased in apparent size on CT at 1–2-month follow-up [61] followed by a gradual decrease in size over the subsequent 1–2 years to volume less than preablation levels. Furthermore, masses > 3 cm^3^ did not increase in apparent size to the same degree as smaller masses, and, in general began to decrease in size immediately following thermal ablation such that at the 1–2-month follow-up larger lesions had decreased to 84% of their original volume.

Dual-energy (DE) CT is finding new uses in assessing thermal ablation. Although not used widely for postprocedure monitoring of HIFU, it has been increasingly explored for use postradiofrequency ablation and thus shows promise in being applied to HIFU. DECT uses photons of different energies (typically 80 and 140 kVp) and can discriminate between material compositions within each voxel when material decomposition is applied. DECT generates more specific information than Hounsfield units; by assuming that each voxel within the image can be decomposed into a water signal and an iodine signal, DECT generates virtual noncontrast (VNC) images and contrast-only (iodine only) images (linearly blended images are often generated to synthesize a traditional Hounsfield unit-based scan). The iodine-only images quantify the distribution of the contrast agent and can delineate margins of thermal ablation. DECT is advantageous since a single acquisition can generate both a VNC image and a blended image, reducing radiation exposure compared to traditional CT which requires pre and postcontrast acquisitions. However, DECT is not a replacement for a dynamic scan. Volumetric iodine concentration, derived from DECT, predicts therapeutic response in preclinical models [74]. In the arterial phase, iodine concentration is greater in residual tumor than surrounding inflammation after 1 week and after 2 weeks for the portal venous phase; at earlier timepoints, the trend may be reversed [75].

Photon-counting CT scanners are now on the horizon and have the potential to improve the assessment of ablation in several ways. First, the spatial resolution is expected to improve due to the smaller size of the photon-counting detector chips as compared with current detectors. Second, the detectors will estimate the energy of each photon, potentially improving spectral CT from 2 to 4 energy levels. Third, the accuracy of the Hounsfield unit estimates is expected to improve. Finally, the radiation dose can potentially be reduced up to 50% [76].

### 4.1. Summary of CT.

CT is not used for real-time monitoring of HIFU procedures clinically, but contrast-enhanced CT is relatively commonly used for postprocedure assessment, with thermally or mechanically ablated tissue presenting as nonperfused regions.

## 5. Ultrasound

### 5.1. Ultrasound for Real-Time Guidance.

**Figure 7:**
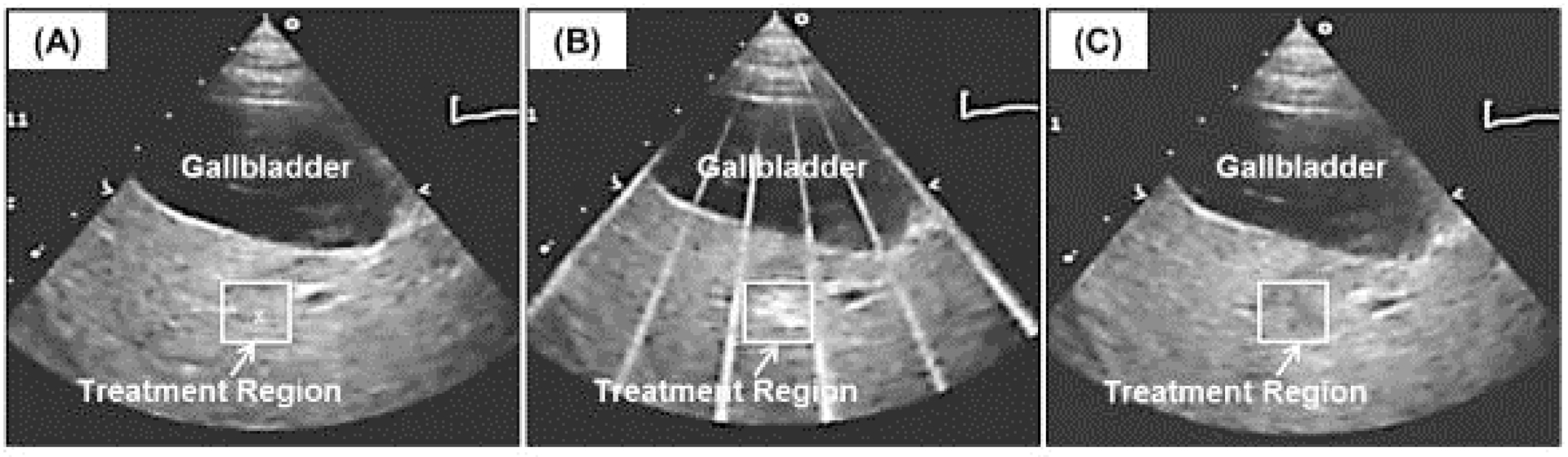
Pre, peri, and postprocedure ultrasound imaging of histotripsy ablation in the liver in a preclinical model. Images show the histotripsy liver treatment monitored by ultrasound imaging before (a), during (b), and after (c) treatment. Prior to each treatment, the bubble cloud was generated in free water with the free-field focal location marked as an “x” on the ultrasound image (a). The histotripsy bubble cloud appears as a dynamically changing hyperechoic region on ultrasound imaging (b) while the resulting lesion appears hypoechoic (c) resulting from fractionation of liver parenchyma. Figure adapted from [19].

Thermal HIFU is not often guided by ultrasound imaging primarily due to the shortcomings of ultrasound thermometry. Due to the nonlinear dependence of the speed of sound and other tissue parameters, ultrasound thermometry cannot be accurately applied at temperatures above ~50°C; thus, accurate measures of thermal dose cannot be obtained during ablative therapies. However, ultrasound guidance of mechanical HIFU, such as histotripsy, has demonstrated good success. In a preclinical model, ultrasound-guided histotripsy of the liver in a porcine model demonstrates that compared to pretreatment imaging (Figure 7(a)), a distinct bubble cloud is observed during treatment (Figure 7(b)). Posttreatment (Figure 7(c)) the mechanically disrupted lesion is hypoechoic. Furthermore, real-time guidance of histotripsy in the clinical setting has recently been reported [10] with good results.

One emerging method that has been utilized to guide ultrasound thermal ablation is quantifying tissue stiffness. Ultrasound methods to assess tissue stiffness have been employed to distinguish between viable and ablated tissue intraprocedurally as well as postablation. The denaturation of proteins and heat-induced dehydration of ablated tissue rapidly alter mechanical properties of tissue. The changes in tissue stiffness can be estimated with ultrasound using acoustic radiation force impulse (ARFI) imaging or shear wave elastography (SWE). US-ARFI imaging has predictive value preablation in uterine fibroids [77]. Ultrasound SWE can provide real-time quantitative estimates of tissue stiffness [78]. US elastography evaluates the extent of tissue necrosis [79–81] via the substantial increase in tissue stiffness following thermal ablation. Thresholding on tissue stiffness correlates with both histopathology and other imaging techniques including contrast-enhanced ultrasound [82] and contrast-enhanced CT [83], but there are reports that US elastography underestimates the extent of ablation [84]. Ultrasound SWE has been used to monitor cardiac [85], liver [86], thyroid [87, 88], and uterine fibroid [89] ablation. However, all ablative procedures have the capacity to generate bubbles through boiling or via cavitation; gas bubbles generated during an ablative procedure can confound elastography measurements [90]. In the presence of bubbles, other techniques such as Nakagami imaging and envelop statistics have been employed with some success to detect ablation-induced bubbles and quantify the ablation zone [91, 92].

In addition to tissue stiffness, contrast-enhanced ultrasound using microbubbles can guide ablation [93, 94] and effectively quantify perfusion postablation [82, 95]. The results correlated well with nonviable tissue on histopathology [96–98], ultrasound elastography [82], and nonperfused volume as measured with contrast-enhanced magnetic resonance imaging (MRI) [99] and CT [100, 101]. Additionally, contrast-enhanced ultrasound has been explored for enhancing the ablative effect of HIFU in preclinical models [6].

### 5.2. Ultrasound for Postprocedure Assessment.

Ultrasound is an inexpensive, real-time, point of care imaging modality, but it has not been as widely employed for post-HIFU monitoring. Ultrasound imaging has been limited by the lack of volumetric imaging on current systems, which makes quantification of morphological markers of response such as RECIST challenging. Although several quantitative ultrasound techniques have been proposed, a move to 3D imaging through large aperture, high channel count arrays would dramatically improve biomarker quantitation.

The wide availability and real-time nature of ultrasound make it well-suited for guiding and assessing interventional treatments. Greyscale b-mode has been explored for peritreatment monitoring and for posttreatment quantification of the ablated volume. On b-mode, the ablated region often appears diffusely hyperechoic [16] and can be differentiated from unablated tissue. However, the hyperechoic characteristics are transient [102]. Transducers integrated within an ablation catheter have demonstrated differing b-mode contrast pre and postablation [103]. However, due to the transient nature of the changes in echogenicity, b-mode is not the most reliable ultrasound technique to assess thermal ablation. Other techniques such as echo decorrelation have been used for real-time monitoring as well as quantitatively measuring thermal lesions [17, 104–106].

**Figure 8:**
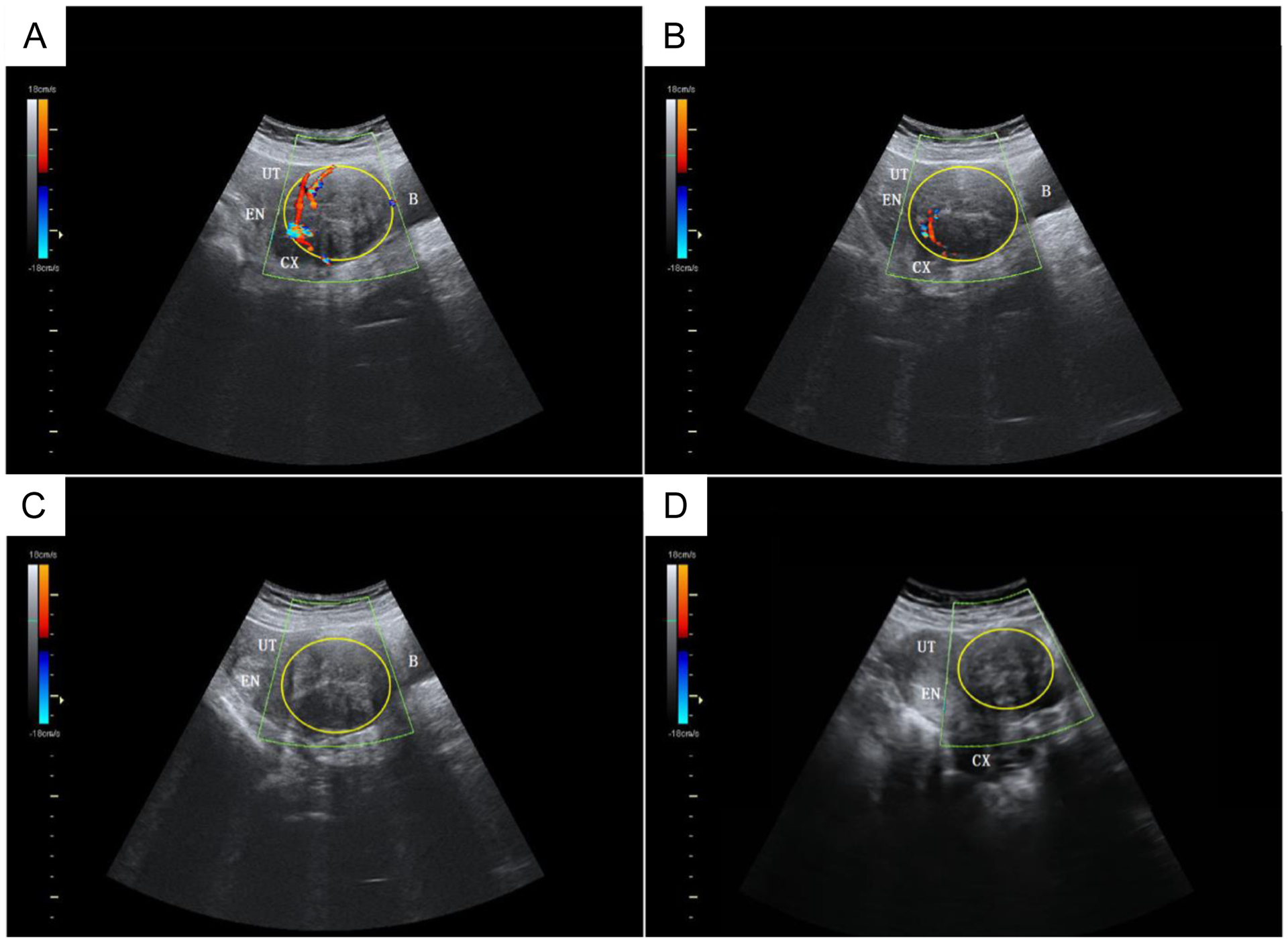
Color Doppler ultrasound imaging pre, peri, and postthermal HIFU ablation. b-mode ultrasound (US) images with color Doppler flow imaging (CDFI) from a 44-y-old patient before and after treatment with thermal HIFU. (a) Pretreatment image shows the vascularity of the fibroid for treatment. (b) Intraoperative assessment result shows that the targeted vessel in the treatment cell was not detected by CDFI and Power Doppler Imaging (PDI). (c) Posttreatment image shows the whole vascularity was not detected by CDFI and PDI. (d) US image with CDFI acquired 3 months after treatment shows fibroid shrinkage. The yellow ring indicates fibroid. B = bladder; CX = cervix; EN = endometrium; UT = uterine. Figure adapted from [18].

Color Doppler ultrasound discriminates between vascularized and nonvascularized tissue and has shown utility in evaluating the nonperfused volume following ablation [18, 107]. In preclinical models, both power Doppler and color Doppler registered a significant decrease in blood flow [16] following HIFU thermal ablation even in the absence of contrast agent. A well vascularized fibroid (Figure 8(a)) as visualized on color Doppler can be monitored intraprocedurally (Figure 8(b)) until vascular flow is no longer observed (Figure 8(c)). Color Doppler can be used in postprocedure follow-up visits (Figure 8(d)) to confirm the absence of tissue regeneration. However, both b-mode and Doppler ultrasound can suffer from overestimation of the ablated volume and have high inter and intraobserver variability [108], making these methods less reliable than other imaging modalities.

### 5.3. Summary of Ultrasound.

Ultrasound is commonly used for real-time monitoring of histotripsy due to its high frame rate and sensitivity to cavitation bubbles. It is not widely employed for thermal HIFU monitoring due to the nonlinearity of US-derived temperature measurements prior to and following tissue coagulation. US is not widely used clinically for postprocedural assessment. However, both color Doppler and contrast-enhanced US are promising methods that may see increasing use clinically as 3D US imaging methods are improved.

## 6. Positron Emission Tomography

While positron emission tomography (PET) is widely used clinically for imaging solid tumors and in providing quantitative measures of response to therapies, it has not been widely used in HIFU ablation assessment. PET imaging involves the injection of a positron-emitting radioisotope, a variety of which are available that can be incorporated into many molecules of biological interest. Due to the comparative lack of background signal, PET has extremely high sensitivity and signal to noise. However, spatial resolution is reduced compared to other modalities. Tracers such as ^18^F-fluorodeoxyglucose (^18^F-FDG) can provide both metabolic and morphological data. As a metabolic marker, ^18^F-FDG PET imaging immediately postablation does not always present with an inflammation-based hyperintense rim around the ablated zone [109] as in other (e.g., CT and MRI) modalities. In liver tumors, a hyperintense rim surrounding the treated region on ^18^F-FDG PET may be indicative of remaining viable tumor as opposed to reflexive hyperemia. Furthermore, a hyperintense rim on ^18^F-FDG PET may develop days to weeks later, but this is due to increased glucose metabolism as tissue regenerates.

**Figure 9:**
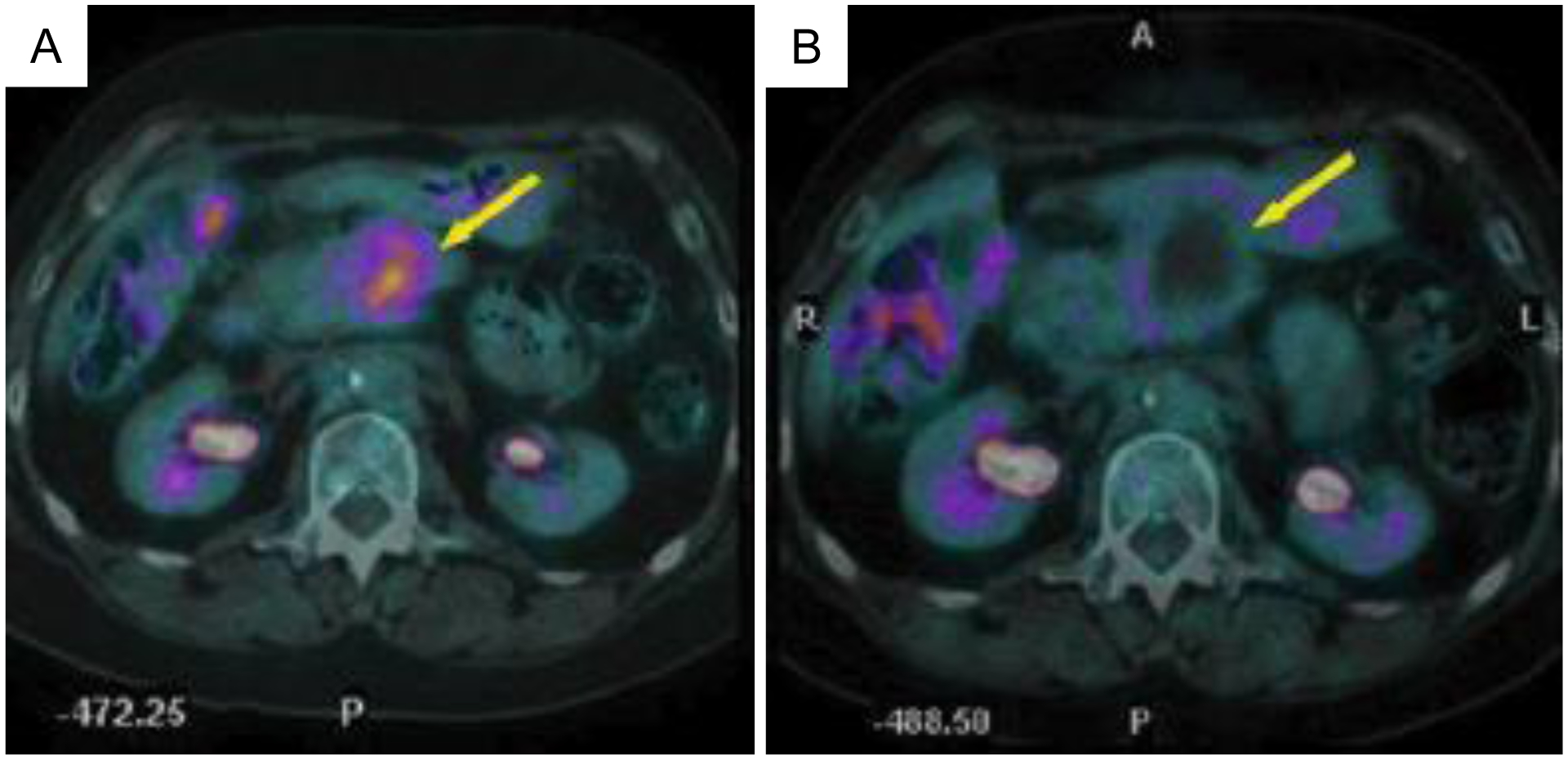
^18^F-FDG PET pre and postthermal HIFU ablation in the pancreas. Transverse view of positron emission tomography computed tomography (PET-CT) images from a 60-year-old patient with pancreatic cancer before and after thermal ablation. (a) Pretreatment PET-CT image showing tumor fluorodeoxyglucose uptake (bold arrow). (b) One month after HIFU, the PET-CT image shows lack of metabolic activity (bold arrow). Figure adapted from [15].

Several studies have suggested ^18^F-FDG PET immediately following ablation can accurately predict treatment efficacy [21, 110], and PET imaging metrics correlate with local disease progression [111] and disease-free survival [112]. Conversely, preablation ^18^F-FDG PET imaging has minimal correlation with local tumor progression and disease-free survival [113]. A meta-analysis of ablative treatments of colorectal liver metastases found 18F-FDG PET to have higher sensitivity (84.6%) than CT (53.4%) for disease progression while both (92.4% for PET and 95.7% for CT) had similar specificities [114]. 18F-FDG PET pre-HIFU (Figure 9(a)) can aid in treatment planning and can be compared with post-HIFU scans (Figure 9(b)) to evaluate the extent of metabolically active tumor.

An emerging area of PET for monitoring and assessing thermal ablation is split-dose ^18^F-FDG PET, which involves preprocedure injection of a small dose of tracer (~1/3 of standard diagnostic dose) followed by a second (~2/3 diagnostic dose) postablation tracer injection. The lower pretreatment dose will have decayed somewhat (~1–1.5 half-life for typical percutaneous ablation) prior to administration of the second dose. A pretreatment image is acquired with the initial dose of tracer and the second dose is used to acquire a second, postablation image. The two acquisitions are registered and used to assess the extent of ablation [112, 115–117] immediately following treatment. The split-dose administration and imaging was developed to over-come the lack of change in avidity of a single preablation dose. The relatively homogeneous uptake of ^18^F-FDG by liver parenchyma makes this method well-suited to assessing the extent of ablative therapies in the liver. However, due to contraction of the tumor during ablation, in some tissues, a split-dose protocol may have trouble discriminating ablation margins [118]. In neuroendocrine liver metastases, split-dose 18F-fluorodihydroxyphenylalanine (^18^F-FDOPA) has been employed for ablation guidance and postablation efficacy with good results in a preliminary clinical study [119].

Another emerging PET technique is combined metabolic and perfusion. This method has not been used in HIFU ablation as yet, but the combination of ^18^F-FDG [120] and ^13^N-ammonia [22] during intraprocedure PET has been explored in liver thermal ablation using microwaves with good results in defining ablation margins. Additionally, ^15^O-water has been evaluated to directly track the behavior of water during repeated thermal dose administrations [121]. The short half-lives of ^13^N (~10 min) and ^15^O (~2 min) allow their repeated use to monitor and delineate ablation margins prior to ending an intervention. While this method has potential application in HIFU ablation given it can quantitatively delineate nonperfused regions, it is most applicable postprocedurally.

**Figure 10:**
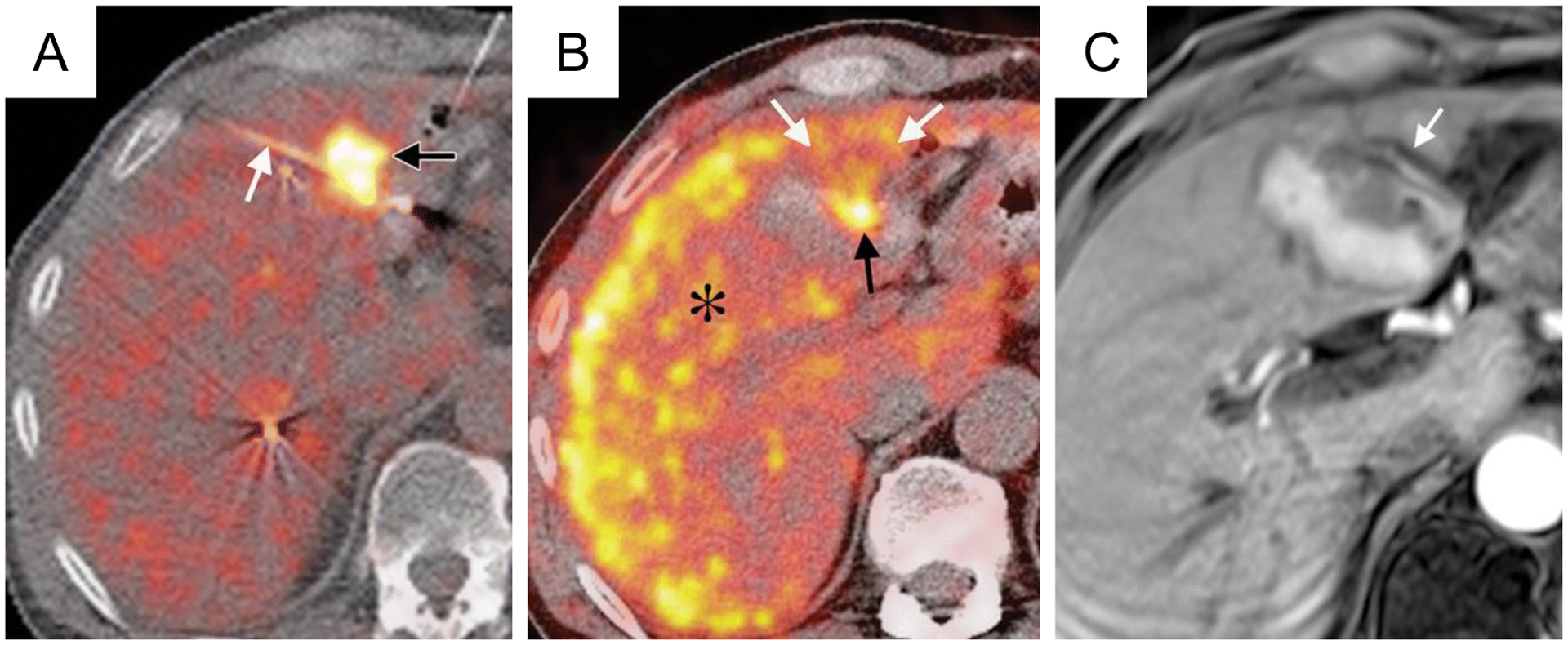
Pre and intraprocedural combined ^18^F-FDG and ^13^N-ammonia PET during microwave ablation compared to contrast-enhanced T1w MRI postprocedure. (a) Fluorodeoxyglucose (FDG) PET/CT image obtained at single breath hold shows FDG uptake in the metastasis (black arrow) and the microwave ablation probe (white arrow) positioned in the posterior half of the tumor. (b) Intraprocedural perfusion PET shown as a fused PET/CT image, with PET image in color, was obtained immediately after microwave ablation. FDG administered before the procedure remains trapped within the tumor (black arrow). Healthy, unablated liver activity (*) is increased by ^13^N-ammonia perfusion. A photopenic ablation margin partially surrounds the ablated tumor from 2 o’clock to 10 o’clock; however, the margin is incomplete anteriorly from 10 o’clock to 2 o’clock (white arrows). (c) Contrast-enhanced T1-weighted MR image acquired 24 hours after the procedure (1.5-T imager; volumetric interpolated brain examination; 4.1/1.6; flip angle, 10°) demonstrates an unsuspected blood vessel (arrow) along the anterior aspect of the ablated tumor; this vascular heat sink likely contributed to the inadequate ablation margin anteriorly. Figure adapted from [22].

In perfusional PET imaging, an initial ^18^F-FDG PET image is acquired prior to treatment (Figure 10(a)) for treatment planning; during the procedure, once ablation is believed to be complete, a short half-life perfusion tracer, such as^13^N-ammonia, is administered and a second image acquired to assess the ablation margins (Figure 10(b)) and compared to postablation contrast-enhanced T1w MRI (Figure 10(c)). The initial FDG uptake by the tumor will be visible. The longer half-life of ^18^F (~110 min) makes re-administration impractical during an interventional procedure; nevertheless, a single perfusion image can be obtained with 18F tracers, which may be sufficient for an end of procedure efficacy assessment. Moreover, ^18^F tracers do not require an on-site cyclotron, increasing their availability.

### 6.1. Summary of PET.

While much less commonly used than MRI and CT, PET is seeing increasing use clinically for postprocedural thermal ablation assessment. Currently, we are not aware of any studies evaluating PET’s ability to assess mechanical HIFU, but tracers that assess perfusion are expected to delineate nonperfused mechanically disrupted regions.

## 7. Conclusion and Future Directions

We have described the common imaging characteristics to guide and assess thermal HIFU and mechanical HIFU on a variety of different modalities. We have also explored emerging technologies in their preclinical stages or in use clinically but applied to other ablative techniques which could be used to assess HIFU ablation. Across all modalities examined, generally, we have found that lesions are nonenhancing very shortly after ablation when perfusion or blood-pool contrast agents are used with the caveat that a hyperemic rim may appear surrounding the ablation zone. This makes it challenging to immediately assess the extent and margins of ablation.

MRI is the most commonly used modality to monitor and assess thermal ablation. Ultrasound is most commonly used for monitoring histotripsy. Assessment of histotripsy ablated volumes has been performed with MRI and CT as well as ultrasound. If multiple intraprocedural assessments are indicated during a thermal ablation procedure, PET tracers that assess perfusion may be preferable to other modalities given the tracer may be administered multiple times safely. However, due to the short half-lives of these tracers, many sites may not have ready access, in which case diffusion-weighted MR imaging would be preferable.

Future extensions of the use of imaging to assess ultrasound-based ablation are likely to incorporate methods to assess the impact of the treatment on the potential to activate an antitumor immune response. Some of the assessment methods currently involve repeated biopsy or blood-based markers such as circulating tumor cells or circulating tumor DNA. The thermal and mechanical effects generated by HIFU can enhance tumor antigen release into the blood and lymph [122–125], and therefore, such assays may be enhanced after ablation. Similarly, immunoPET methodologies have been developed to assess changes in local immune cell numbers and activation and readers are referred to excellent reviews for further information on such techniques [126, 127].

## Figures and Tables

**Table 1:** Summary of selected high intensity focused ultrasound (HIFU) studies and imaging findings across multiple modalities. We summarize a selection of imaging findings following both thermal and mechanical HIFU ablation. Two radiofrequency ablation (RFA) studies are also included due to novelty of the imaging methods and its applicability to thermal HIFU assessment.

	Study	Ablation Modality	Imaging Modality	Clinical/ Pre-clinical	Findings
MRI	Hoogenboom et al.^5^	HIFU (boiling histotripsy)	Non-contrast T2 MRI	Pre-clinical	Ablated region is hyperintense with hypointense rim on noncontrast T2w MRI
	Rouviere et al.^7^	HIFU (thermal)	Contrast enhanced T1 MRI	Clinical	Ablated region is non-enhancing accompanied (variably) with a hyperintense rim due to hyperemia
	Pilatou et al.^8^	HIFU (thermal)	Diffusion weighted and ADC MRI	Clinical	Ablated lesions have increased intensity on high b value DWI images
	Liao et al.^9^	HIFU (thermal)	Diffusion weighted MRI	Clinical	Reduction in ADC following ablation; high-signal ring around ablated region on DWI
	Vidal-Jove et al.^10^	HIFU (histotripsy)	Contrast- enhanced T1 MRI	Clinical	Mechanically disrupted region appears as a non-perfused volume
	Morochnik et al.^11^	HIFU (thermal)	T2 map MRI	Clinical	T2 increases during HIFU treatment
	Worlikar et al.^12^	HIFU (histotripsy)	T2w MRI	Pre-clinical	Ablated region hypointense immediately following ablation followed by gradual resorption
	Lee et al.^13^	HIFU (thermal)	^13^C MRI	Pre-clinical	Ablated regions exhibited ^13^C lactate and ^13^C urea intensity similar to background. Partially ablated regions had decreased intensity which recovered after 5 days
CT	Yang et al.^14^	HIFU (thermal)	Contrast- enhanced CT	Pre-clinical	Ablated lesion is low-density and non-enhancing
	Orgera et al.^15^	HIFU (thermal)	Contrast- enhanced CT	Clinical	Non-enhancing ablated lesion appears shortly after ablation
US	Luo et al.^16^	HIFU +/− microbubbles	US	Pre-clinical	Transient hyperechogenicity after ablation on b-mode
	Sasaki et al.^17^	HIFU (thermal)	US	Pre-clinical	Decorrelation between RF frames during HIFU
	Zhou et al.^18^	HIFU (thermal)	Doppler US	Clinical	No blood flow on Doppler after HIFU ablation
	Vlaisavljevich et al.^19^	HIFU (histotripsy)	US	Pre-clinical	Bubble cloud can be used to monitor treatment in real time
PET	Chen et al.^20^	RFA	^18^FDG PET	Pre-clinical	PET had increased sensitivity (94.1%) and specificity (81.3%) to recurrent tumor after thermal ablation
	Orgera et al.^15^	HIFU (thermal)	^18^FDG PET	Clinical	Reduction in metabolic activity after HIFU delineates ablation margins
	Shyn et al.^21^	RFA	^13^N ammonia and ^18^FDG PET	Clinical	Entire ablation margin can be assessed intraprocedurally
